# Enhanced flux pinning in YBCO multilayer films with BCO nanodots and segmented BZO nanorods

**DOI:** 10.1038/s41598-017-13758-6

**Published:** 2017-10-31

**Authors:** Mika Malmivirta, Hannes Rijckaert, Ville Paasonen, Hannu Huhtinen, Teemu Hynninen, Rajveer Jha, Veerpal Singh Awana, Isabel Van Driessche, Petriina Paturi

**Affiliations:** 1 Wihuri Physical Laboratory, Department of Physics and Astronomy, University of Turku, FI-20014, Turku, Finland; 2 University of Turku Graduate School (UTUGS), University of Turku, FI-20014, Turku, Finland; 3 SCRiPTS, Department of Inorganic and Physical Chemistry, Ghent University, Krijgslaan 281 S3, 9000 Ghent, Belgium; 4 Superconductivity Division, National Physical Laboratory (CSIR), New Delhi, 110012 India

## Abstract

The flux pinning properties of the high temperature superconductor YBa_2_Cu_3_O_7−*δ*_ (YBCO) have been conventionally improved by creating both columnar and dot-like pinning centres into the YBCO matrix. To study the effects of differently doped multilayer structures on pinning, several samples consisting of a multiple number of individually BaZrO_3_ (BZO) and BaCeO_3_ (BCO) doped YBCO layers were fabricated. In the YBCO matrix, BZO forms columnar and BCO dot-like defects. The multilayer structure improves pinning capability throughout the whole angular range, giving rise to a high critical current density, *J*_c_. However, the BZO doped monolayer reference still has the most isotropic *J*_c_. Even though BZO forms nanorods, in this work the samples with multiple thin layers do not exhibit a *c* axis peak in the angular dependence of *J*_c_. The angular dependencies and the approximately correct magnitude of *J*_c_ were also verified using a molecular dynamics simulation.

## Introduction

To tune the high-temperature superconductor YBa_2_Cu_3_O_7−*δ*_ (YBCO) towards higher critical current, *J*_c_, and smaller anisotropy, it has been found useful to create defects of various sizes and shapes into the YBCO matrix. Commonly, YBCO is doped with a non-superconducting dopant, like BaZrO_3_^1,2^ (BZO) or BaSnO_3_^3,4^ (BSO). More recently also Ba_2_YNbO_6_^5^ has been used and doping can be fine-tuned by additionally using Ba_2_YTaO_6_^6^. All these dopants create nanosized rods when deposited using pulsed laser deposition (PLD). However, YBCO has also been doped using materials that result in non-correlated pinning centres, like Y_2_O_3_^7^ or BaCeO_3_^8,9^ (BCO). Especially the nanorods are seen to decrease anisotropy and they commonly produce a peak in the angular dependence of *J*_c_ when the magnetic field *B* is parallel to the YBCO *c* axis and the nanorods. Furthermore, different kinds of dopants have been seen to affect intrinsic properties like the coherence length of YBCO^10,11^.

Especially in PLD made films, non-correlated, nanosized dot-like defects are rarely used alone, but combined with nanorods for enhanced pinning. For example combinations of Y_2_O_3_ nanodots and BZO nanorods^12^ or Y_2_O_3_ nanodots and BSO nanorods^13^ have successfully been used. As described in the work by Horide *et al*.^13^, the amount of dopant can affect the shape of the angular dependence of *J*_c_ drastically. Thus, for the best result, application-related design is needed. In addition to mixing different types of dopants, multilayer structures containing layers of BZO doped YBCO^14,15^ or BSO doped YBCO^16,17^ along with layers of undoped YBCO have also been studied. The segmented nanorods that are formed by depositing undoped YBCO between the layers of doped YBCO provide larger pinning force especially in the direction of **B||** *ab* plane of YBCO, as compared to the continuous nanorods. In that direction, the ends of the nanorods actually pin stronger than any other defect^16^. Furthermore, by tuning the length of the segmented nanorods, the behaviour of *J*_c_ in different magnetic fields and orientations can be greatly modified. Because of their structure, the segmented nanorods can act as more diverse pinning centres than rods penetrating through the whole sample. Despite the promising results in multilayers, to our knowledge only Liu and Du^18^ have utilized the pinning possibilities of the spacer layer between the nanorod layers.

A multilayer is a complex pinning landscape and to be able to deeply understand how the vortices move, a computational model is needed. Calculations are usually based on solving Ginzburg-Landau equations^19,20^ where all the interactions come into the model intrinsically but these models are computationally expensive. Another way to study vortices is to use molecular dynamics (MD) simulations^21,22^ to describe effective vortex dynamics. However, such models have previously been used only as toy models for studying the critical dynamics of vortex flow. To our knowledge, MD models that can reproduce the angular dependence of *J*_c_, have not been published.

In this paper, a set of multilayer films were made using layers of equal thicknesses of BZO and BCO doped YBCO. The number and thickness of each layer were varied and their effect on the angular dependence of *J*_c_ was measured. Additionally, the movement of vortices in such structures was studied in detail by using a molecular dynamics simulation capable of anisotropy scaling.

## Results and Discussion

### Structural properties

**Figure 1:**
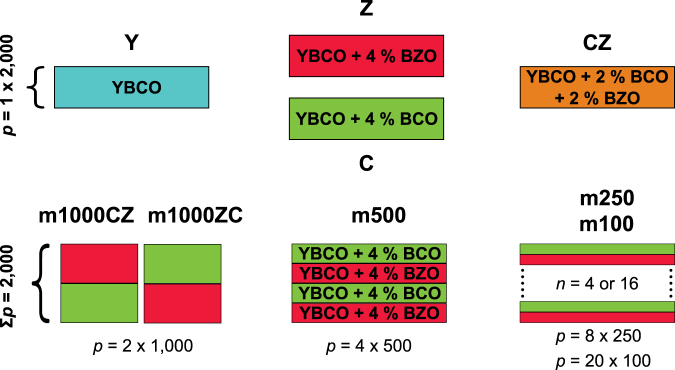
Schematic diagram of the samples that were made. In the figure, *p* is the number of pulses and *n* the number of layers.

Films with alternating layers of BZO and BCO doped layers were fabricated with pulsed laser deposition. The different multilayer structures are schematically shown in Fig. 1. The films are all free from impurities, as confirmed by the X-ray diffraction (XRD) *θ*−2*θ* measurements. There is no clear correlation between the layer thickness and the *c* parameter, although both BCO and BZO doping increases the *c* lattice parameter slightly (see Supplementary Information (SI) for more details). The smallest strain in the *c* direction is in the Y and Z samples but there are no large discrepancies between different multilayer samples.

**Figure 2:**
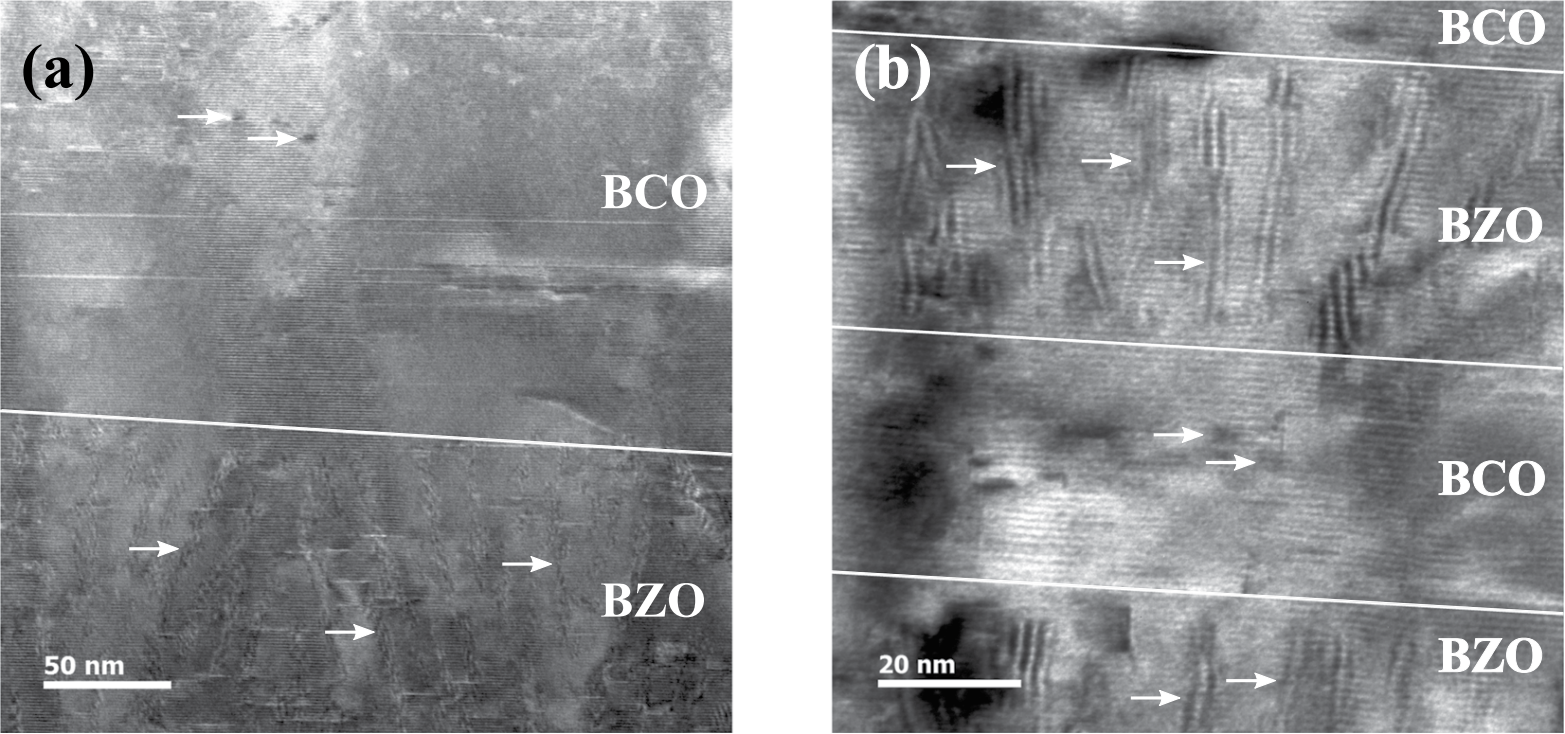
The TEM images of (**a**) m1000ZC and (**b**) m250 showing the interfaces between nanorods and nanodots. The arrows indicate some of the rods in the BZO doped layer and some of the nanodots in the BCO doped layer. The solid lines show the transition from BZO doped layer into BCO doped one and vice versa.

The high resolution transmission electron microscopy (HRTEM) images of m1000ZC, m250 and m100 (see Fig. 1 for sample names) all show nanorods in BZO doped layers and spherical particles in BCO doped layers. As shown on the HRTEM image of m1000ZC (Fig. 2(a)), the nanorods are splayed and flexural with a mutual average distance of (10 ± 1) nm and diameter (5 ± 1) nm. The BCO layer, on the other hand, contains small BCO particles, (3 ± 1) nm in diameter. The nanorods in m250 (Fig. 2(b)) have similar diameter and distance as compared to m1000ZC, although the rods are shorter due to the smaller thickness of the layer. Furthermore, it is remarkable that the nanorods in m250 have not grown on top of each other, unlike e.g. the rods in BSO doped YBCO rods with undoped YBCO in between^16^. The self-aligned growth has been attributed to strain fields created by the dopant^23^ and since BCO itself tends to increase strain in YBCO^8,9^, it likely disturbs the rod formation process. Additionally, in m100 the thickness and distance of the BZO nanorods is the same as in samples with thicker layers but the rods are straight.

### Superconducting properties

The resistively measured *T*_c_ s are roughly between 87.7 K and 88.6 K, without a clear correlation with the number of the layers. Only CZ has *T*_c_ 85.0 K which reflects the distortions in the structure. The distortions are also clearly visible in the XRD and TEM measurements of CZ. The width of the transition, Δ *T*_c_, defined as the difference in temperatures corresponding to 80% and 10% of the normal state resistance, is roughly 2 K for all other but 1.4 K for Z and 3.9 K for CZ. This means that there is no degradation of superconductivity due to layer structure, and only CZ has a distorted structure.

**Figure 3:**
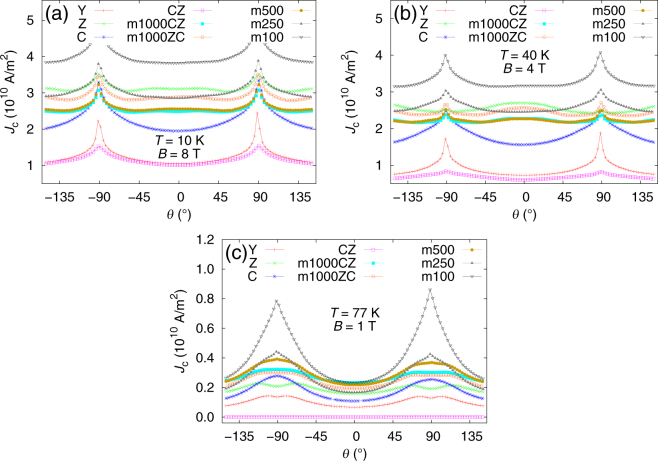
The angular dependence of the samples at (**a**) 10 K and 8 T, (**b**) 40 K and 4 T and (**c**) 77 K and 1 T. The *c* peak can be seen around *θ* = 0°. A part of the data for m100 at 10 K is missing due to current limitations of the measurement system.

The angular dependencies of *J*_c_ at 40 K and 4 T are highly dependent on the number of layers (Fig. 3(b)). The 40 K is selected to be far enough from the *T*_c_ so that the small differences in *T*_c_ do not have an effect. On the other hand, the 40 K temperature is more important from a technological point of view than 10 K. Because of the current limitation in the measurement system, the 40 K also allows measurement of all samples even at lower fields. The 4 T magnetic field enables to probe the differences in vortex-vortex interaction. Samples with long enough nanorods exhibit a *c* axis peak, i.e. as **B||** *c* axis of YBCO (later referred as *c* peak) which corresponds to *θ* = 0°. As the layer thickness is decreased, the *c* peak is not seen any more. This limit is between m500 and m250. Thus, the absence of *c* peak does not imply the absence of *c* axis correlated defects. The disappearance of the *c* peak^24^ can be explained using the vortex path model. It states that if the vortex goes along one pinning site in the *c* direction less than the standard deviation of the sideways step of the vortices, the peak disappears. Similar disappearance of the *c* peak has been seen on non-continuous BZO nanorods^25^ and on BSO doped YBCO films with a multilayer structure of undoped YBCO in between^16^. However, as Matsumoto *et al*.^16^ pointed out, the ends of the nanorods are powerful for pinning in the *ab* direction.

The angular dependencies of *J*_c_ at 10 K show similar properties to 40 K (Fig. 3(a)). Still, the BZO doped Z has one of the most isotropic angular dependencies. Also, it can be seen that the *c* peak of Z and m1000CZ and m1000ZC are very wide, with a double peak structure. It originates from the tilt and flexural nature of the nanorods. The tilt can be adjusted by fine-tuning the deposition temperature^25^. The order of the curves is pretty similar to situation at 40 K and thus there are no large differences in the pinning capabilities of the samples at these temperatures.

At 77 K, 1 T (Fig. 3(c)) the situation is different. No sample has a *c* peak but shoulders evolve near the *ab* peak. Still m100 has the highest *J*_c_ values throughout all angles. The *c* peak disappears due to the thermal fluctuations of vortices and larger size of the vortices and they both affect the paths the vortices make^24^. The CZ has practically zero *J*_c_, likely due to the wide transition and low *T*_c_.

Furthermore, it can be seen from Fig. 3 that, within this series, the absolute value of *J*_c_ is highest on m100. This is true at all measured temperatures at low fields but also at low temperatures and high fields. The Y and CZ samples have lower *J*_c_ s, but for all the other samples, *J*_c_ s are very close to each other and the differences can be accounted to statistical sample-to-sample variation. The comparison is done only within this sample series because the actual *J*_c_ can depend on the critical voltage as well as on the way the measurements are made^26^. Similarly, enhanced *J*_c_ is also possible to achieve using surface pinning^27^ but also using a multilayer structures without any doping^28,29^. Samples with many layers are kept at the deposition temperature for a longer time. This allows the structure to be more relaxed compared to samples deposited within minutes. In multilayer samples containing YBCO and NdBa_2_Cu_3_O_7−*δ*_^28^ (NdBCO) layers, it was seen that the longer time at the deposition temperature allows the interface to relax. However, the added local stress due to lattice mismatch between the layers is relieved as out-of-plane edge dislocations. Even if the NdBCO is replaced by YBCO^29^, so that there is only a waiting times during the deposition, as if depositing a true multilayer, improvements in *J*_c_ are seen. In the multilayer samples of the current study, it is seen that the BCO doped layer is more strained than the BZO layer and thus the transition from BCO to BZO doped layer does not happen smoothly at every point due to the strain. This could probably trigger similar out-of-plane edge dislocations in the YBCO structure as seen in literature^28,29^. In general, it is suggested that interfacial dislocations are responsible for the increase of the self-field *J*_c_^30^.

Also, because the nanorods in the current work have not grown on top of each other, they can act as a dense network capable of pinning a large number of vortices. This is in contrast to a work by Matsumoto *et al*.^16^ where the nanorods grew on top of each other but no increase in *J*_c_ throughout all the angles was seen with thinner layers. On the contrary, in our work at low fields the short nanorods can provide strong enough pinning centres for the small number of vortices present in the sample. The BZO pinning sites together with BCO nanodots in the spacer layer prevent the vortices from creeping from one pinning site into another by preventing the kink from sliding along the rod. Creeping would cost extra because it is energetically unfavourable for a vortex to leave the dot pinning site. If the spacer layer was undoped YBCO, this hopping by kink formation would be more likely to happen because there would not be pinning sites in the spacer layer with pinning force comparable to large BZO rods. Additionally, at higher magnetic field tilted from the **B||** ***c***, the vortices have a large number of possibilities to be pinned by the short nanorods. As the field is tilted further, the vortices still like to remain in the same pinning sites since partial hopping into the next pinning site would cost too much in energy. When the field angle is large enough and hopping occurs, the segmented nanorod structure will allow the vortices to align themselves along the external field. This is in contrast to the BZO doped sample which provides only straight and continuous nanorods^2^. Additionally, the BCO particles between the rod layers can provide some extra pinning^9^ as compared to works with multilayer architecture having undoped YBCO between the nanorod layers, although the different alignment of the nanorods in these cases may also affect the strength of the pinning^16,17^.

**Figure 4:**
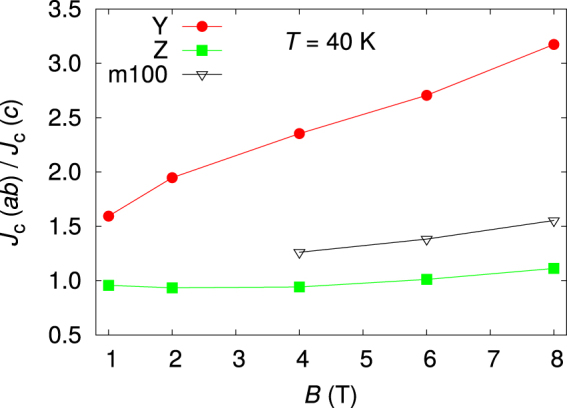
The ratio *J*_c_ (*ab*)/*J*_c_ (*c*) at 40 K on selected samples.

Because of the existence of the *c* peak, the Blatter scaling type anisotropy determination could not be introduced, as was done for example in refs^9,31^. Instead, to describe the anisotropy of the films, the ratio *J*_c_ (*ab*)/*J*_c_ (*c*) was calculated at 40 K and is shown for Y, Z and m100 in Fig. 4. Z gives the lowest ratio, m100 has the highest absolute *J*_c_ and Y is the reference. The ratio for Y increases above 3 in 8 T field. For m100 the ratio is lower than that, although there is no *c* peak. This is due to enhanced flux pinning, but it is also noteworthy that the absolute value is higher throughout all angles. Hence, the same absolute drop in *J*_c_ gives a smaller ratio because of the higher absolute *J*_c_. The value for the most isotropic sample, Z, is rather close to one in all fields, and by tuning the deposition temperature of the film, the ratio could be decreased even further^25^. However, because of the *c* peak, the sample is not completely isotropic as the ratio would suggest, but the *J*_c_ is lower on the angles between the peaks. Nevertheless, by tuning suitable pinning centres, the real anisotropy of the samples can be greatly affected, as can also be seen for example by looking at the *J*_c_(*θ*) data in the work by Horide *et al*.^13^.

## Simulation results

A molecular dynamics simulation was made to describe the movement of vortices in different pinning landscapes. The simulation takes into account vortex-vortex interaction^22,32^, vortex-pinning site interaction^33^ and line tension^34^ due to vortex line energy. Furthermore, the magnetic energy of the vortex in magnetic field was also included and vortices were also subject to Lorentz force due to current in the superconductor. Also, a drag-like force^35^ was taken into account. Additionally, where applicable, the Blatter scaling^34^ was used to enable to use the simulation with tilted fields with respect to the *c* axis. In the model, the mass of the vortices is an adjustable parameter which do not affect the results as long as the time step is short enough.

**Figure 5:**
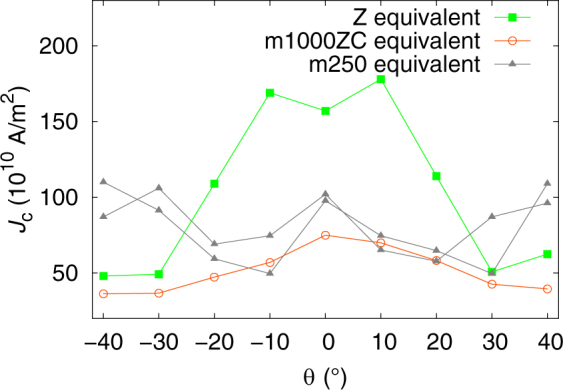
The angular dependence of the modelling results with different pinning landscapes at the field of 0.75 T. The “m250 equivalent” has been plotted using two slightly modified pinning landscapes. The *c* peak can be seen around *θ* = 0°.

The simulations reproduce the shape of *J*_c_(*θ*) around the **B||** *c* axis of YBCO but also the approximately correct magnitude of *J*_c_ without any scaling or fitting. A *c* peak can be observed when the pinning landscape consists only of straight nanorods (Fig. 5), but a similar peak is also observed using a structure similar to m1000ZC. When using a structure of layers of nanorods and nanodots repeated 4 times, like in m250, no *c* peak is seen. The simulation for”m250 equivalent” has been made with two different, slightly modified pinning landscapes in order to probe the effect of structural randomness. However, there is not enough statistics to determine whether the narrow *c* peak at *θ* = 0° is an artefact or not. Experimentally a small *c* peak has been observed e.g. in undoped YBCO films with a large number of dislocations^36^. Additionally, the absolute value of *J*_c_ is larger in the rod pinning landscape than in the multilayer samples. This suggests that there are also other pinning centres in the real multilayer samples, for instance strain and dislocations, that have an effect on *J*_c_. However, the effect of nanoinclusions on pinning still dominates^37,38^.

**Figure 6:**
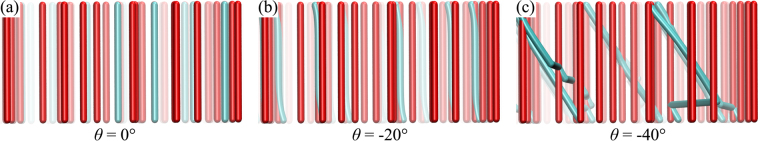
Vortices at stable state in the “Z equivalent” sample at *J*_c_. The pictures are taken at the same place. The magnetic field is rotated (**a**) 0° (**b**) −20° (**c**) −40° with respect to the direction of the nanorods. The nanorods are red and vortices turquoise. The density of the vortices corresponds to a 0.75 T field. The simulation results were visualized using VMD^43^.

Figure 6 shows vortices in the steady state of the simulation at *J*_c_ as the pinning landscape consists of nanorods. The current is at the critical limit and thus the picture can be used to explain the *c* peak. Strong nanorods are able to pin the vortices almost along their entire length even at *θ* = −20°, and the vortices are turned parallel to the nanorods. However, that is not possible any more with *θ* = −40° and nanorods act more like point pinning centres. This results in the decrease of the *J*_c_ value. The high Lorentz force due to high current causes the vortices in tilted angles to be longer. In such case the line tension of a vortex is not strong enough to keep it as short as possible.

**Figure 7:**
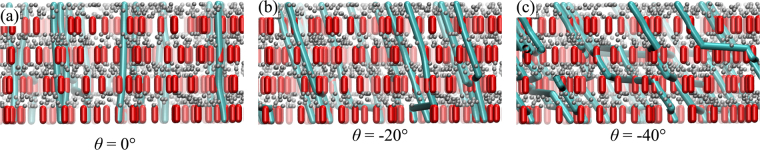
Vortices at stable state in the “m250 equivalent” sample at *J*_c_. The pictures are taken at the same place. The magnetic field is rotated (**a**) 0° (**b**) −20° (**c**) −40° with respect to the direction of the nanorods. The Lorentz force is directed out from the figure. The nanorods are red, point pinning sites grey and vortices turquoise. The density of the vortices corresponds to a 0.75 T field.

In the”m250 equivalent” case the situation is similar when the field is tilted. Figure 7 shows the vortices at the critical current limit in the stable state. At *θ* = 0° the vortices follow especially nanorods. To better describe the real, observed situation, the nanorods have not been put on top of each other but they have a small shift. This causes partly the short nanorods to act more like point pinning centres already at zero angle and not even in this case do the vortices go entirely along the nanorod segments but tend to bend away earlier. At *θ* = −20° the vortices start to jump more from one pinning site into another and the number of jumps is even larger at *θ* = −40°. The nanorods are less and less capable to pin the vortices along their direction. Furthermore, it seems that, although the nanodots are capable to pin, their effect is remarkably smaller than that of the nanorods. However, the model may underestimate the effect of nanodots, since they also cause strain in the lattice^9^ and that would increase the pinning force in real samples.

The evolvement into stable configuration at *θ* = −20° can also be seen as an animation at the web page of SI in two pinning landscapes, nanorods (Animation S1) and”m250 equivalent” (Animation S2). For clarity, the periodic pinning landscapes have been expanded. In the animations, the *J*_c_ flows from right to left and thus the Lorentz force drives the vortices towards the viewer.

## Conclusions

In conclusion, YBCO multilayer films with BZO and BCO dopants were fabricated. The film with only BZO nanorods produces the most isotropic *J*_c_(*θ*). On the other hand, a film with a large number of layers of BZO and BCO doped YBCO has a higher absolute value of *J*_c_. The short BZO nanorods along with the BCO nanodot layers form a structure that makes it too expensive to move for a vortex. Additionally, even though BZO is columnar even in the thinnest layers, the nanorods act more like nanodots and there is no *c* peak in the *J*_c_(*θ*). However, on samples with thick enough BZO layers, the *c* peak can be seen. The disappearance of *c* peak in the case of short nanorods was also confirmed using a molecular dynamics simulation. Thus, if there is no *c* peak in the angular dependence of the *J*_c_ it cannot be said that the sample does not contain columnar defects of any kind. From the applications point of view, if isotropic *J*_c_ is needed, a sample with continuous nanorods is the best choice of those presented in this paper. On the other hand, in power applications, where a high *J*_c_ is needed, a multilayer structure would work better.

## Methods

A set of multilayer samples was made using pulsed laser deposition with 2,000 pulses per sample. Both the BCO and BZO doped targets contain 4 wt-% of dopant. Also, for comparison, a target with both 2 wt-% BCO and 2 wt-% BZO was introduced. In the films, the number and thickness of bilayers, BZO doped YBCO combined with BCO doped YBCO, was varied. A set of multilayer samples were made on 5 × 5 mm^2^ SrTiO_3_ (001) substrates using pulsed laser deposition system (*λ* = 308 nm). The pulse duration was 25 ns and the repetition rate was set on 5 Hz with a laser fluence of 1.3 J/cm^2^. The samples were deposited at 750° C and after that oxygenated at 700° C for 10 minutes. The changing of the deposition target in multilayer samples was done manually *in situ*. Thus the time it took at 750° C to make a single layer film, like Z, was considerably shorter than the time needed to deposit m100, for example. The BZO and the undoped YBCO targets were made using sol-gel method^39,40^, implying nanosized grains in the targets and BCO doped using solid state reaction^8^, implying micron sized grains. The target containing both 2 wt-% BCO and 2 wt-% BZO was also made using solid state reaction.

The phase purity and crystalline properties of the samples were studied by XRD using a Philips X’Pert Pro MPD with a texture goniometer. Samples CZ, m1000ZC, m250 and m100 were further studied using CS-corrected HRTEM. The used equipment for TEM analysis was FEI Nova 600 Nanolab Dual Beam FIB-SEM for TEM preparation and a JEOL JEM 2200-FS TEM, operated at 200 kV, for imaging. For the TEM measurements, a cross-sectional lamella was cut via the FIB *in situ* lift out procedure with an Omniprobe extraction needle and top cleaning^41^.

For the angular dependence measurements, a 50 *μ*m wide current stripe, having the voltage lead separation of 1.86 mm, was patterned onto samples using wet chemical etching. After etching, the dimensions of the current stripe were measured using a Bruker Innova atomic force microscope. The contacts for the resistive measurements were made by tapping with indium. The critical current measurements as a function of angle between magnetic field and the sample were made using the horizontal rotator probe of a Quantum Design Physical property measurement system. The criterion for *I*_c_ was 215 *μ*V/cm. However, the high value does not change the shape of the angular dependencies^42^.

The datasets generated during and/or analysed during the current study are available from the corresponding author on reasonable request.

## Electronic supplementary material

Supplementary information

Supplementary Animation S1

Supplementary Animation S2

## References

[1] MacManus-Driscoll JL (2004). Strongly enhanced current densities in superconducting coated conductors of Yba_2_Cu_3_O_7−*x*_ +BaZrO_3_. Nat. Mater..

[2] Goyal A (2005). Irradiation free, columnar defects comprised of self-assembled nanodots and nanorods resulting in strongly enhanced flux pinning in Yba_2_Cu_3_O_7−δ_ films. Supercond. Sci. Technol..

[3] Varanasi CV (2006). Flux pinning enhancement in Yba_2_Cu_3_O_7−*x*_ films with BaSnO_3_ nanoparticles. Supercond. Sci. Technol..

[4] Varanasi CV (2007). Enhancement and angular dependence of transport critical current density in pulsed laser deposited Yba_2_Cu_3_O_7−*x*_ BaSnO_3_ films in applied magnetic fields. J. Appl. Phys..

[5] Feldmann DM (2010). Improved flux pinning in YBa_2_Cu_3_O_7_ with nanorods of the double perovskite Ba_2_YnbO_6_. Supercond. Sci. Technol..

[6] Opherden L (2016). Large pinning forces and matching effects in YBa_2_Cu_3_O_7−δ_ thin films with Ba_2_Y(Nb/Ta)O_6_ nano-precipitates. Sci. Rep..

[7] Mele P (2007). Insertion of nanoparticulate artificial pinning centres in YBa_2_Cu_3_O_7−*x*_ films by laser ablation of a Y_2_O_3_-surface modified target. Supercond. Sci. Technol..

[8] Irjala M, Huhtinen H, Jha R, Awana VPS, Paturi P (2011). Optimization of the BaCeO_3_ concentration in YBCO films prepared by pulsed laser deposition. IEEE Trans. Appl. Supercond..

[9] Malmivirta M (2015). The angular dependence of critical current of BaCeO_3_ doped Yba_2_Cu_3_O_6+*x*_ thin films. IEEE Trans. Appl. Supercond..

[10] Palonen H, Huhtinen H, Shakhov MA, Paturi P (2013). Electron mass anisotropy of BaZrO_3_ doped YBCO thin films in pulsed magnetic fields up to 30 T. Supercond. Sci. Technol..

[11] Malmivirta M (2016). Dirty limit scattering behind the decreased anisotropy of doped YBa_2_Cu_3_O_7−δ_ thin films. J. Phys. Cond. Mat..

[12] Maiorov B (2009). Synergetic combination of different types of defect to optimize pinning landscape using BaZrO_3_-doped Yba_2_Cu_3_O_7_. Nat. Mater..

[13] Horide T (2013). *J*_c_ improvement by double artificial pinning centers of BaSnO_3_ nanorods and Y_2_O_3_ nanoparticles in Yba_2_Cu_3_O_7_ coated conductors. Supercond. Sci. Technol..

[14] Ichinose A (2007). Microstructures and critical current densities of YBCO films containing structure-controlled BaZrO_3_ nanorods. Supercond. Sci. Technol..

[15] Kiessling A (2011). Nanocolumns in Yba_2_Cu_3_O_7−*x*_/BaZrO_3_ quasi-multilayers: formation and influence on superconducting properties. Supercond. Sci. Technol..

[16] Matsumoto K (2014). Irreversibility fields and critical current densities in strongly pinned Yba_2_Cu_3_O_7−*x*_ films with BaSnO_3_ nanorods: The influence of segmented BaSnO_3_ nanorods. J. Appl. Phys..

[17] Horide T (2016). Hybrid artificial pinning centers of elongated-nanorods and segmented-nanorods in Yba_2_Cu_3_O_7_ films. Supercond. Sci. Technol..

[18] Liu Y, Du G (2011). Preparation and flux-pinning properties of multilayered yttrium barium copper oxide thin films containing alternating barium zirconate and yttria nanostructures. Journal of Electronic Materials.

[19] Palonen H, Jäykkä J, Paturi P (2012). Modeling reduced field dependence of critical current density in Yba_2_Cu_3_O_6+*x*_ films with nanorods. Phys. Rev. B.

[20] Sadovskyy IA (2016). Toward superconducting critical current by design. Advanced Materials.

[21] Dobramysl U, Assi H, Pleimling M, Täuber UC (2013). Relaxation dynamics in type-II superconductors with point-like and correlated disorder. The European Physical Journal B.

[22] Assi H, Chaturvedi H, Dobramysl U, Pleimling M, Täuber UC (2016). Disordered vortex matter out of equilibrium: a Langevin molecular dynamics study. Molecular Simulation.

[23] Xie Q, Madhukar A, Chen P, Kobayashi NP (1995). Vertically self-organized InAs quantum box islands on GaAs(100). Phys. Rev. Lett..

[24] Paturi P (2010). The vortex path model and angular dependence of *J*_c_ in thin YBCO films deposited from undoped and BaZrO_3_-doped targets. Supercond. Sci. Technol..

[25] Malmivirta M (2014). Three ranges of the angular dependence of cirtical current of BaZrO_3_ doped Yba_2_Cu_3_O_7−*δ*_ thin films grown at different temperatures. Thin Solid Films.

[26] Pan AV, Golovchansky IA, Fedoseev SA (2013). Critical current density: Measurement vs. reality. Europhysics Letters.

[27] Khokhlov VA (2004). Surface pinning as origin of high critical current in superconducting films. Supercond. Sci. Technol..

[28] Pan AV, Pysarenko SV, Dou SX (2006). Drastic improvement of surface structure and current-carrying ability in yba_2_cu_3_o_7_ films by introducing multilayered structure. Appl. Phys. Lett..

[29] Pan AV, Pysarenko SV, Wexler D, Rubanov S, Dou SX (2007). Multilayering and ag-doping for properties and performance enhancement in yba_2_cu_3_o_7_ films. IEEE Trans. Appl. Supercond..

[30] Foltyn SR (2007). Materials science challenges for high-temperature superconducting wire. Nat. Mater..

[31] Llordés A (2012). Nanoscale strain-induced pair suppression as a vortex-pinning mechanism in high-temperature superconductors. Nat. Mater..

[32] Brandt EH (1986). Elastic and plastic properties of the flux-line lattice in type-II superconductors. Phys. Rev. B.

[33] Pan V (2006). Supercurrent transport in Yba_2_Cu_3_O_7−*δ*_ epitaxial thin films in a dc magnetic field. Phys. Rev. B.

[34] Blatter G, Feigel’man MV, Geshkenbein VB, Larkin AI, Vinokur VM (1994). Vortices in high-temperature superconductors. Reviews of Modern Physics.

[35] Bardeen J, Stephen MJ (1965). Theory of the motion of vortices in superconductors. Phys. Rev..

[36] Gutiérrez J, Puig T, Obradors X (2007). Anisotropy and strength of vortex pinning centers in Yba_2_Cu_3_O_7−*x*_ coated conductors. Appl. Phys. Lett..

[37] Matsumoto K, Mele P (2010). Artificial pinning center technology to enhance vortex pinning in YBCO coated conductors. Supercond. Sci. Technol..

[38] Paturi P, Malmivirta M, Palonen H, Huhtinen H (2016). Dopant diameter dependence of *J*_c_(B) in doped YBCO films. IEEE Trans. Appl. Supercond..

[39] Peurla M (2006). Optimization of the BaZrO_3_ concentration in YBCO films prepared by pulsed laser deposition. Supercond. Sci. Technol..

[40] Raittila J, Huhtinen H, Paturi P, Stepanov YP (2002). Preparation of superconducting Yba_2_Cu_3_O_7−*δ*_ nanopowder by deoxydation in Ar before final oxygenation. Physica C.

[41] Montoya E, Bals S, Rossell MD, Schryvers D, Tendeloo GV (2007). Evaluation of top, angle, and side cleaned FIB samples for TEM analysis. Microscopy Research and Technique.

[42] Durrell JH, Feldmann DM, Cantoni C (2007). Suppression of vortex channeling in meandered Yba_2_Cu_3_O_7−*δ*_ grain boundaries. Appl. Phys. Lett..

[43] Humphrey W, Dalke A, Schulten K (1996). VMD - visual molecular dynamics. J. Molec. Graphics.

