# Supplementary material for: Enhanced flux pinning in YBCO multilayer films with BCO nanodots and segmented BZO nanorods
Source: Sci Rep. 2017 Oct 31;7:14682. doi: 10.1038/s41598-017-13758-6 (PMC5665862; doi:10.1038/s41598-017-13758-6)
Supplement: Supplementary file 1 — Supplementary information [file 41598_2017_13758_MOESM1_ESM.pdf]

Supplementary Information: Enhanced flux pinning in YBCO multilayer films with BCO nanodots and segmented BZO nanorods

Mika Malmivirta[1,2,*], **Hannes Rijckaert**[3], **Ville Paasonen**[1], **Hannu Huhtinen**[1], **Teemu Hynninen**[1], **Rajveer Jha**[4], **Veerpal Singh Awana**[4], **Isabel Van Driessche**[3], **and Petriina Paturi**[1]

[1]Wihuri Physical Laboratory, Department of Physics and Astronomy, University of Turku, FI-20014 Turku, Finland
[2]University of Turku Graduate School (UTUGS), University of Turku, FI-20014 Turku, Finland.
[3]SCRiPTS, Department of Inorganic and Physical Chemistry, Ghent University, Krijgslaan 281 S3, 9000 Ghent, Belgium
[4]Superconductivity Division, National Physical Laboratory (CSIR), New Delhi 110012, India
[*]mika.malmivirta@utu.fi

XRD characterizations

The phase purity was checked by measuring $\theta - 2\theta$ scans and no impurities were found (Fig. S1). The strongest peak of BZO is seen around 43° which confirms the crystallinity of BZO. Furthermore, the BCO (002) is seen around 41° but since the particles are very small,[S1,S2] only a wide and weak peak is seen around that value. Furthermore, the measurement was used to define the *c* axis parameter of YBCO (Table S1). The dopant increases the length of the *c* axis, 11.68 Å on the undoped sample and at least 11.71 Å on all the doped samples. Since there is no clear correlation with the number of layers in the film, it is highly possible that the differences between samples other than the undoped Y are caused by insignificant sample-to-sample variation. The correlation length[S3] (Table S1) in the samples, signifying the order of the lattice, is between 80 and 100 Å, only Y and Z are better ordered. This seems to be in line with the FWHMs of the YBCO (005) as well, that are below 0.30° on all samples, but Y and Z have significantly smaller FWHM, 0.15° and 0.10°, respectively. Furthermore, according to the $2\theta - \phi$ scans of YBCO (212), the films are biaxially twinned, apart from the CZ which has a distorted twin structure. The scan of YBCO (102) showed that there were no *a* axis oriented grains.

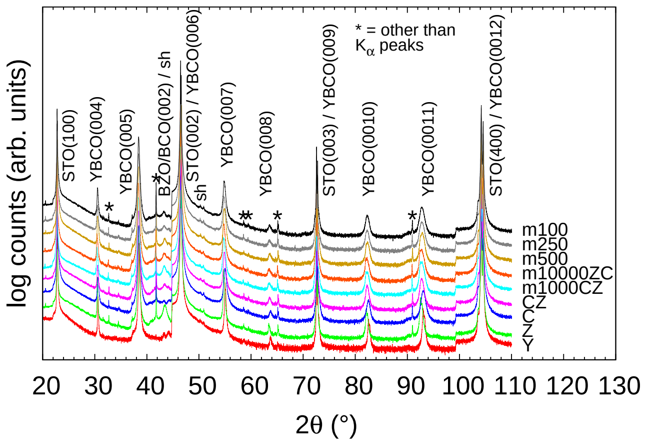

Figure S1. The $\theta - 2\theta$ measurements of all samples.

Table S1. The most important structural parameters measured by XRD and the resistive critical temperatures, T_c and the widths of the transitions, ΔT_c.

sample	FWHM YBCO (005) (°)	*c* axis length (Å)	r_c (Å)	T_c (K)	ΔT_c (K)
Y	0.15	11.680	124	88.4	1.4
C	0.28	11.722	74	87.5	2.1
Z	0.10	11.727	154	87.8	2.2
CZ	0.26	11.729	76	85.0	3.9
m1000CZ	0.29	11.719	88	87.5	1.9
m1000ZC	0.29	11.730	96	88.1	2.0
m500	0.29	11.713	98	88.4	1.9
m250	0.29	11.724	88	88.0	2.0
m100	0.25	11.721	95	88.4	1.9

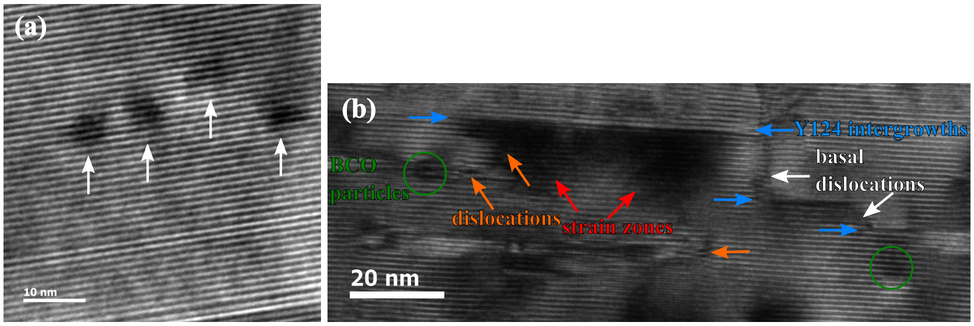

Figure S2. (a) The BCO particles seen in the m1000ZC sample, indicated by white arrows. (b) Other defects in the same sample seen in the TEM images.

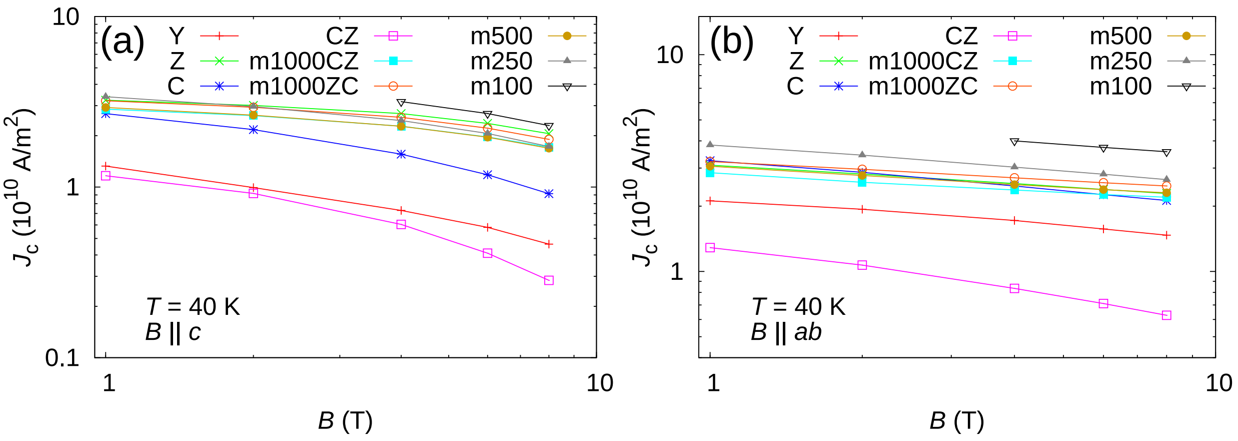

Figure S3. The critical current densities at 40 K (a) in the case $B||c$ axis of YBCO and (b) $B||ab$ plane, extracted from the angular dependencies. The part of the data for m100 is missing due to current limitations of the measurement system.

TEM characterizations

The TEM image (Fig. S2 (a)) shows BCO particles in m1000ZC. The diameter of the particles is roughly (3±1) nm. Other defects seen in the BCO layer of 1000ZC (Fig. S2 (b)) are strained zones, dislocations, basal dislocations and Y124 intergrowths. Additionally, the BCO layer contains very long stacking faults that are seen as white lines in the images. The BZO layer has also stacking faults, although shorter ones. The interface between the BZO layer and the BCO doped layer is good, although there are some dislocations to accommodate strain changes between a BCO and BZO doped layers. On the STO substrate/film interface, there is a 15 – 20 nm thick layer that is not fully crystallized. On the BZO/BCO interface, there can be seen accumulation of BCO, sometimes above BZO nanorods. As a whole, the effect of these defects on pinning is smaller as compared to correlated pinning centres.[S4,S5]

As measured by TEM, the thickness of m250 sample is 310 nm, somewhat less than the 380 nm of the m1000ZC. The m250 has a 6 nm disordered layer at the film/substrate interface. The nanorods in m250 are similar to 1000ZC, although shorter because of the smaller layer thickness. The twin regions are small which means that there are also correspondingly large number of twin boundaries. The sample m100 is 305 nm thick with a 5 nm layer of unordered YBCO next to the substrate interface. The BZO here is not splayed, but straight and the BZO layer thickness is on average only 16 nm. The thickness and distance of the nanorods is the same as on samples with thicker layers. The CZ sample, on the other hand, has both good and strained areas, and altogether the sample is very strained. There are no BZO columns but some nanodots have formed nanocolumn-like features.

Superconducting properties

To get a good picture of the pinning properties of the samples, the measurements of $J_c(\theta)$ were made at 10, 40 and 77 K in 1, 2, 4, 6 and 8 T. The magnetic field dependencies of J_c at 40 K, extracted from the angular data, show clear differences between samples. In the case $B||c$ axis of YBCO the (Fig. S3 (a)), the samples having a c peak due to BZO have a high J_c within the deposited samples. Also, samples with nanorods but no c peak have a rather high J_c value. Only the m250 is deteriorated more in increasing field than other samples with similar self-field J_c. In the ab direction (Fig. S3 (b)) the Z sample with a monolayer of BZO is not any more among the highest J_c samples. Here, especially the high J_c values of samples with a large number of layers can be most clearly seen. Only the J_c of the C sample decreases faster under the external magnetic field than other samples with similar value of J_c in the self-field.

Simulation details

All vortices in the simulation were divided into 40 vertical parts, i.e. the simulation was formed of stacked layers. All the layers were subject to periodic boundary conditions in the ab plane. Thus the vortices leaving the samples due to the Lorentz force re-enter from the other side. Inside each layer, a vortex can interact with other vortices in the same layer. Between the layers, the line tension of a vortex acts as a binding force. Because of the layered structure, the simulation cannot be used to describe the situations close to case $B||ab$ plane of YBCO. The only defects that were introduced to pin vortices were either nanorods (radius of 3 nm) or nanodots (radius 1.5 nm). The distribution of the nanorods was taken from previous

experiments[S6] but for computational reasons their mutual distances were doubled. If there were another layer of nanorods, like in ”m250 equivalent” sample, the position of the nanorods at another layer was varied slightly. This was done to avoid having nanorods on top of each other, as suggested by the experimental results in this work. The positions of the nanodots were random. No other typical defects for YBCO were introduced because we wanted to see the effect of these defects in particular. The defects were divided so that there are first nanorod layers with certain thickness then equally many nanodot layers. This was repeated until the total thickness of simulation is 40 layers. To speed up the stabilization of the vortex lattice, initially the vortices were set into hexagonal lattice.[S7] In the model, the temperature is not implemented i.e. it is 0 K. The simulation was written in Python using molecular dynamics with velocity Verlet algorithm.

The J_c was iterated by bisection method. The simulation was run with one current and the next current was adjusted according to the stability of the found state. The solution was considered stable if the vortices were moving less than double the coherence length of YBCO in the *ab* plane. This was done by comparing the position of the vortex at 1,000 and 500 steps earlier with the present coordinates. Also, if this condition of stability was not fulfilled, the stability was determined based on the speed of vortices. If it was below 200 m/s, the simulation was considered stable. Both stability conditions were checked at the same time, with regular intervals.

References

S1. Malmivirta, M. *et al.* The angular dependence of critical current of $BaCeO_3$ doped $YBa_2Cu_3O_{6+x}$ thin films. *IEEE Trans. Appl. Supercond.* **25**, 6603305 (2015).

S2. Malmivirta, M. *et al.* Dirty limit scattering behind the decreased anisotropy of doped $YBa_2Cu_3O_{7-\delta}$ thin films. *J. Phys. Cond. Mat.* **28**, 175702 (2016).

S3. Gauzzi, A. & Pavuna, D. Quantitative analysis of growth-induced reduction of long range lattice order in ion-beam sputtered $YBa_2Cu_3O_{6.9}$ films. *Appl. Phys. Lett.* **66**, 1836–1838 (1995).

S4. Matsumoto, K. & Mele, P. Artificial pinning center technology to enhance vortex pinning in YBCO coated conductors. *Supercond. Sci. Technol.* **23**, 014001 (2010).

S5. Paturi, P., Malmivirta, M., Palonen, H. & Huhtinen, H. Dopant diameter dependence of $J_c(B)$ in doped YBCO films. *IEEE Trans. Appl. Supercond.* **26**, 8000705 (2016).

S6. Peurla, M. *et al.* Optimization of the $BaZrO_3$ concentration in YBCO films prepared by pulsed laser deposition. *Supercond. Sci. Technol.* **19**, 767–771 (2006).

S7. Blatter, G., Feigel’man, M. V., Geshkenbein, V. B., Larkin, A. I. & Vinokur, V. M. Vortices in high-temperature superconductors. *Rev. Mod. Phys.* **66**, 1125–1388 (1994).
